# Blockade of PD-L1/PD-1 signaling promotes osteo-/odontogenic differentiation through Ras activation

**DOI:** 10.1038/s41368-022-00168-2

**Published:** 2022-04-01

**Authors:** So Mi Jeon, Je Sun Lim, Su Hwan Park, Hyung Joon Kim, Hyung-Ryong Kim, Jong-Ho Lee

**Affiliations:** 1 Department of Health Sciences, The Graduate School of Dong-A University, Busan, Republic of Korea; 2 Department of Oral Physiology, Periodontal Diseases Signaling Network Research Center, Dental and Life Science Institute, School of Dentistry, Pusan National University, Yangsan, Republic of Korea; 3 Department of Pharmacology, College of Dentistry, Jeonbuk National University, Jeonju, Republic of Korea; 4 Department of Biomedical Sciences, Dong-A University, Busan, Republic of Korea

**Keywords:** Stem-cell differentiation, Cell signalling

## Abstract

The programmed cell death ligand 1 (PD-L1) and its receptor programmed cell death 1 (PD-1) deliver inhibitory signals to regulate immunological tolerance during immune-mediated diseases. However, the role of PD-1 signaling and its blockade effect on human dental pulp stem cells (hDPSCs) differentiation into the osteo-/odontogenic lineage remain unknown. We show here that PD-L1 expression, but not PD-1, is downregulated during osteo-/odontogenic differentiation of hDPSCs. Importantly, PD-L1/PD-1 signaling has been shown to negatively regulate the osteo-/odontogenic differentiation of hDPSCs. Mechanistically, depletion of either PD-L1 or PD-1 expression increased ERK and AKT phosphorylation levels through the upregulation of Ras enzyme activity, which plays a pivotal role during hDPSCs osteo-/odontogenic differentiation. Treatment with nivolumab (a human anti-PD-1 monoclonal antibody), which targets PD-1 to prevent PD-L1 binding, successfully enhanced osteo-/odontogenic differentiation of hDPSCs through enhanced Ras activity-mediated phosphorylation of ERK and AKT. Our findings underscore that downregulation of PD-L1 expression accompanies during osteo-/odontogenic differentiation, and hDPSCs-intrinsic PD-1 signaling inhibits osteo-/odontogenic differentiation. These findings provide a significant basis that PD-1 blockade could be effective immunotherapeutic strategies in hDPSCs-mediated dental pulp regeneration.

## Introduction

Human dental pulp stem cells (hDPSCs) are rich in human dental pulp tissues.^1^ Recently, hDPSCs have been substantially considered for several applications in the regenerative medicine and tissue engineering fields due to their benefits over the other sources. First, they are easily accessible and are easily obtained during routine dental procedures without ethical concerns, and can retain their stem cell properties even after long cryopreservation.^2^ Second, hDPSCs have a great potential with multilineage differentiation; they can differentiate into odontogenic lineage that express gene-specific markers, such as runt-related transcription factor 2 (RUNX2), alkaline phosphatase (ALP), dentin matrix protein-1 (DMP-1), and dentin sialophosphoprotein (DSPP).^3^ Several hDPSCs transplantation studies have shown regenerative capacity of a dentin/pulp-like structure in animal models.^1,4–6^ Therefore, understanding the biological mechanisms of hDPSC differentiation is essential for providing successful hDPSCs engineering strategies in dental pulp therapy.

The programmed cell death 1 (PD-1) receptor-mediated negative immune signaling is well reported in a wide range of immune-mediated diseases, such as acute/chronic infections and cancers by regulating T cell effector functions.^7^ PD-1 is expressed in many types of immune cells, including activated T cell, and PD-1-induced negative immune signaling is mediated by engaging its ligands-the programmed cell death 1 ligand 1 (PD-L1; encoded by *CD274*) and/or PD-L2 (encoded by *CD273*), which result in T cell exhaustion.^7^ It is well reported that extracellular interaction between PD-L1 on tumor cells and PD-1 on CD8^+^ T cells induces the suppression of CD8^+^ T cell proliferation and function, and the promotion of CD8^+^ T cell apoptosis, allowing tumor escape from the immune surveillance within the tumor microenvironment.^8,9^ These findings have been successfully translated to the clinical applications and made a huge effect on cancer therapy; treatment with PD-1 blocking antibodies has shown success in enhancing durable anti-tumor immune responses, and has consequently been approved by the US Food and Drug Administration (FDA) in cancer therapy.^10–13^ However, it remains unknown the role of PD-L1 and PD-1 in hDPSCs differentiation into the odontogenic lineage or whether PD-1 blockade affects odontogenic differentiation.

Ras expression is ubiquitously detected in many tissues where they regulate diverse cellular functions.^14^ In humans, the three distinct Ras isoforms, namely, H-Ras, N-Ras, and K-Ras, are encoded by corresponding genes.^14,15^ Ras proteins act as binary molecular switches that cycle between inactive guanosine diphosphate (GDP)-bound and active guanosine triphosphate (GTP)-bound states to regulate cellular processes.^15^ The guanine nucleotide exchange-factors (GEFs) catalyzes conversion of inactive form to the active form. Although Ras has an intrinsic GTPase activity that facilitates the deactivation of active proteins to inactive state, the deactivation process is accelerated by GTPase-activating proteins (GAPs). Thus, GEFs and GAPs mediate the activation/deactivation cycle of the Ras proteins.^15,16^ Upon activation, GTP-bound active Ras proteins undergo a conformational change in their switch domains, allowing them to recruit a large set of Ras effector proteins such as RAF kinase and phosphoinositide 3-kinase (PI3K).^15,16^ Since the Ras effector proteins have a putative Ras binding domain (RBD), Ras proteins physically associate with the RAF kinase or PI3K to activate the RAF/MEK/ERK or PI3K/AKT cascades, respectively, which regulate diverse cellular responses.^15^ Therefore, Ras proteins are important regulators of multiple normal cell physiology aspects as well as malignant transformation. Although Ras proteins are involved in many biological processes, their role and regulation in odontogenic differentiation remain unknown.

In the present study, we investigated (1) the functional roles of PD-L1/PD-1 and Ras in the odontogenic differentiation of hDPSCs, (2) whether PD-L1/PD-1 signaling functioned through modifying Ras protein activities, and (3) the possible application of PD-1 blockade in promoting odontogenic differentiation of hDPSCs.

## Results

### PD-L1 expression is downregulated during the hDPSCs differentiation into the osteo-/odontogenic lineage

**Fig. 1:**
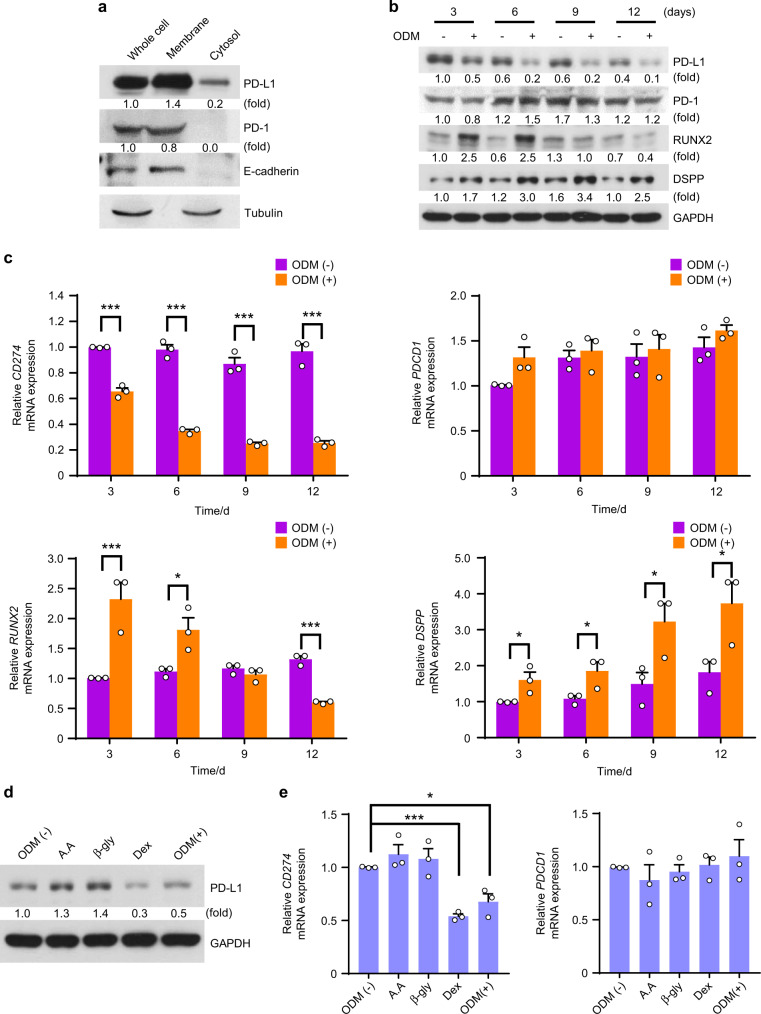
PD-L1 expression is downregulated during hDPSCs differentiation into the osteo-/odontogenic lineage. **a** hDPSCs were harvested for the isolation of membrane and cytosolic fractions. Immunoblotting analyses were carried out and representative band intensity was quantified. **b** and **c** hDPSCs were cultured with or without ODM for the indicated days. Immunoblotting analyses were carried out and representative band intensity was quantified (**b**). Real-time PCR data for *CD274*, *PDCD1*, *RUNX2*, and *DSPP* (**c**). *P < 0.05; **P < 0.01; ***P < 0.001, Student’s *t* test. **d** and **e** hDPSCs were treated with or without A.A (l-ascorbic acid 2-phosphate), β-gly (β-glycerol phosphate), Dex (dexamethasone), or ODM for one day. Immunoblotting analyses were carried out and representative band intensity was quantified (**d**). Real-time PCR data for *CD274* and *PDCD1* (**e**). *P < 0.05; ***P < 0.001, Student’s *t* test.

We first checked whether hDPSCs express immune checkpoint proteins PD-L1 and PD-1, and showed that hDPSCs express both PD-L1 and PD-1 (Fig. 1a, b). Fractionation analyses of hDPSCs revealed that PD-L1 was localized in both cell membrane and cytoplasm, whereas PD-1 was localized only in the cell membrane (Fig. 1a). Next, we assessed the expressional patterns of PD-L1 and PD-1 during hDPSCs differentiation into the osteo-/odontogenic lineage. hDPSCs were cultured with an osteo-/odontogenic differentiation medium (ODM), which consisted of l-ascorbic acid 2-phosphate, β-glycerol phosphate, and dexamethasone in a-MEM complete medium, for the indicated days (Fig. 1b, c). The ODM stimulation successfully induced the expression of RUNX2 and DSPP (Fig. 1b, c). Interestingly, we found that PD-L1 expression, not PD-1, was downregulated during hDPSCs differentiation into the osteo-/odontogenic lineage, compared to their unstimulated control (Fig. 1b, c). To evaluate which factors are involved in the PD-L1 downregulation, hDPSCs were treated with l-ascorbic acid 2-phosphate, β-glycerol phosphate, or dexamethasone for one day. PD-L1 downregulation was observed only in dexamethasone-treated hDPSCs, but not in l-ascorbic acid 2-phosphate- or β-glycerol phosphate-treated (Fig. 1d, e). In contrast, PD-1 expression was not altered by dexamethasone (Fig. 1e). These results suggest that hDPSCs express both PD-L1 and PD-1, and only PD-L1 is downregulated during ODM-induced hDPSCs differentiation into the osteo-/odontogenic lineage.

### PD-L1 inhibits hDPSCs differentiation into the osteo-/odontogenic lineage

**Fig. 2:**
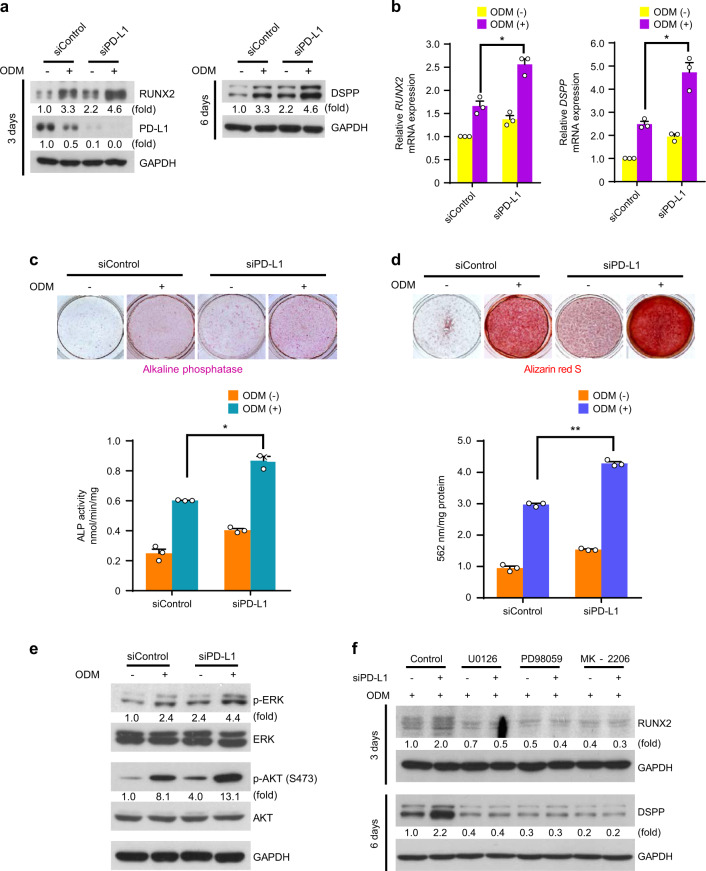
PD-L1 inhibits hDPSCs differentiation into the osteo-/odontogenic lineage. **a** and **b** The control siRNA or PD-L1 siRNA-transfected hDPSCs were cultured with or without ODM for 3 days (for PD-L1 and RUNX2) or 6 days (for DSPP). Immunoblotting analyses were carried out and representative band intensity was quantified (**a**). Real-time PCR data for *RUNX2* and *DSPP* (**b**). *P < 0.05, two-way ANOVA test. **c** and **d**. The control siRNA or PD-L1 siRNA-transfected hDPSCs were cultured with or without ODM for 6 days (for ALP staining and activity assay) or 21 days (for Alizarin red S staining and quantification). ALP staining and activity assay were performed (**c**). Alizarin red S staining was performed and quantified (**d**). *P < 0.05; **P < 0.01, two-way ANOVA test. **e** The control siRNA or PD-L1 siRNA-transfected hDPSCs were cultured with or without ODM for one day. Immunoblotting analyses were carried out and representative band intensity was quantified. **f** The control siRNA or PD-L1 siRNA-transfected hDPSCs were cultured with ODM or the indicated inhibitors (U0126, 10 μmol·L^−1^; PD98059, 10 μmol·L^−1^; or MK-2206, 5 μmol·L^−1^) for three days (for RUNX2) or six days (for DSPP). Immunoblotting analyses were carried out and representative band intensity was quantified.

We next explored the potential role of PD-L1 in hDPSCs differentiation into the osteo-/odontogenic lineage using short interfering RNA (siRNA) to knockdown endogenous *CD274*, which encodes PD-L1. The PD-L1 siRNA treatment largely decreased protein expression levels of PD-L1 in hDPSCs (Fig. 2a). The knockdown of PD-L1 expression resulted in increased basal and ODM-induced protein and mRNA levels of RUNX2 and DSPP (Fig. 2a, b), ALP activities (Fig. 2c), and ability to form mineralized nodules (Fig. 2d). These results indicate that PD-L1 expression inhibits the hDPSCs differentiation into the osteo-/odontogenic lineage.

We previously reported that ODM stimulation induces the ERK and PI3K/AKT, and p38 in hDPSCs,^17^ which play an essential role in the hDPSCs differentiation into the osteo-/odontogenic lineage (Fig. S1). Therefore, we checked whether these signals are affected by PD-L1 expression. As shown in Fig. 2e, PD-L1 knockdown markedly increased basal and ODM-induced phosphorylation levels of ERK and AKT, but not p38 (data not shown), in hDPSCs. Moreover, treatment of ERK inhibitor U0126 or PD98059 or AKT inhibitor MK-2206 abolished the PD-L1 silencing-induced expression levels of differentiation markers (Fig. 2f). Together, these results demonstrate that PD-L1 expression negatively regulates the hDPSCs differentiation into the osteo-/odontogenic lineage *via* downregulation of the ERK and AKT signals.

### PD-1 inhibits hDPSCs differentiation into the osteo-/odontogenic lineage

**Fig. 3:**
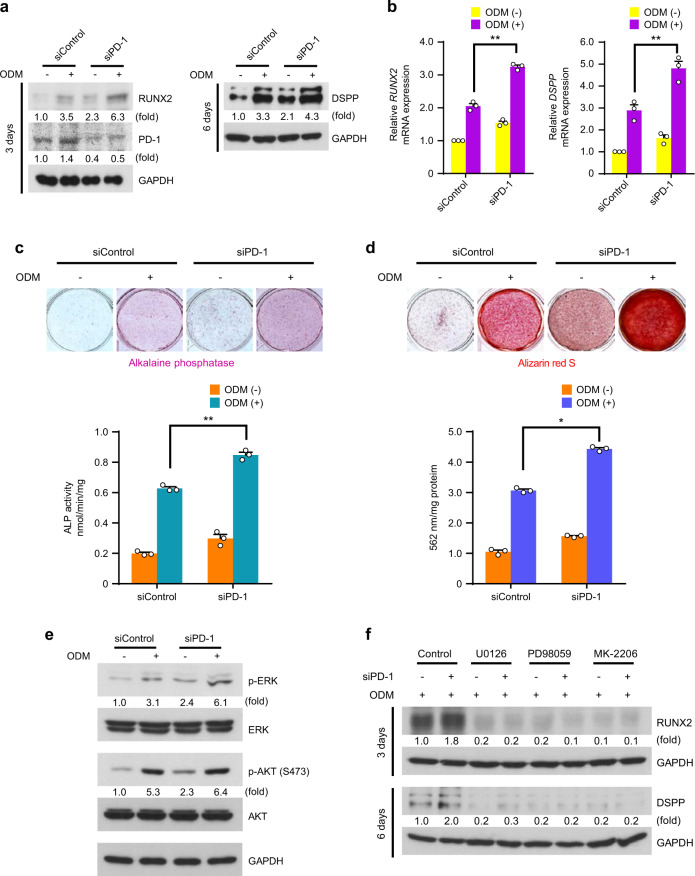
PD-1 inhibits hDPSCs differentiation into the osteo-/odontogenic lineage. **a** and **b** The control siRNA or PD-1 siRNA-transfected hDPSCs were cultured with or without ODM for 3 days (for RUNX2) or 6 days (for DSPP). Immunoblotting analyses were carried out and representative band intensity was quantified (**a**). Real-time PCR data for *RUNX2* and *DSPP* (**b**). **P < 0.01, two-way ANOVA test. **c** and **d** The control siRNA or PD-1 siRNA-transfected hDPSCs were cultured with or without ODM for 6 days (for ALP staining and activity assay) or 21 days (for Alizarin red S staining and quantification). ALP staining and activity assay were performed (**c**). Alizarin red S staining was performed and quantified (**d**). *P < 0.05; **P < 0.01, two-way ANOVA test. **e** The control siRNA or PD-1 siRNA-transfected hDPSCs were cultured with or without ODM for one day. Immunoblotting analyses were carried out and representative band intensity was quantified. **f** The control siRNA or PD-1 siRNA-transfected hDPSCs were cultured with ODM or the indicated inhibitors (U0126, 10 μmol·L^−1^; PD98059, 10 μmol·L^−1^; or MK-2206, 5 μmol·L^−1^) for 3 days (for RUNX2) or 6 days (for DSPP). Immunoblotting analyses were carried out and representative band intensity was quantified.

Both PD-L1 and PD-1 were localized in the cell membrane, although the expressional patterns were different during hDPSCs differentiation into the osteo-/odontogenic lineage (Fig. 1). Therefore, we hypothesized that PD-1 may have similar effects to those of PD-L1 on hDPSCs differentiation into the osteo-/odontogenic lineage. We also used siRNA to target endogenous *PDCD1*, which encodes PD-1. The PD-1 siRNA treatment successfully decreased expression levels of PD-1 in hDPSCs (Fig. 3a and Fig. S2). PD-1-depleted hDPSCs showed increased basal and ODM-induced protein and mRNA levels of RUNX2 and DSPP (Fig. 3a, b), ALP activities (Fig. 3c), and ability to form mineralized nodules (Fig. 3d). In addition, PD-1 knockdown increased basal and ODM-induced phosphorylation levels of ERK and AKT, but not p38 (data not shown), in hDPSCs (Fig. 3e). Inhibition of the PD-1 depletion-activated ERK and AKT signals resulted in blocked expression levels of differentiation markers (Fig. 3f). These results demonstrate that PD-1 expression also negatively regulates the hDPSCs differentiation into the osteo-/odontogenic lineage *via* downregulation of the ERK and AKT signals.

### Silencing of PD-L1/PD-1 promotes hDPSCs differentiation into the osteo-/odontogenic lineage *via* Ras activation

**Fig. 4:**
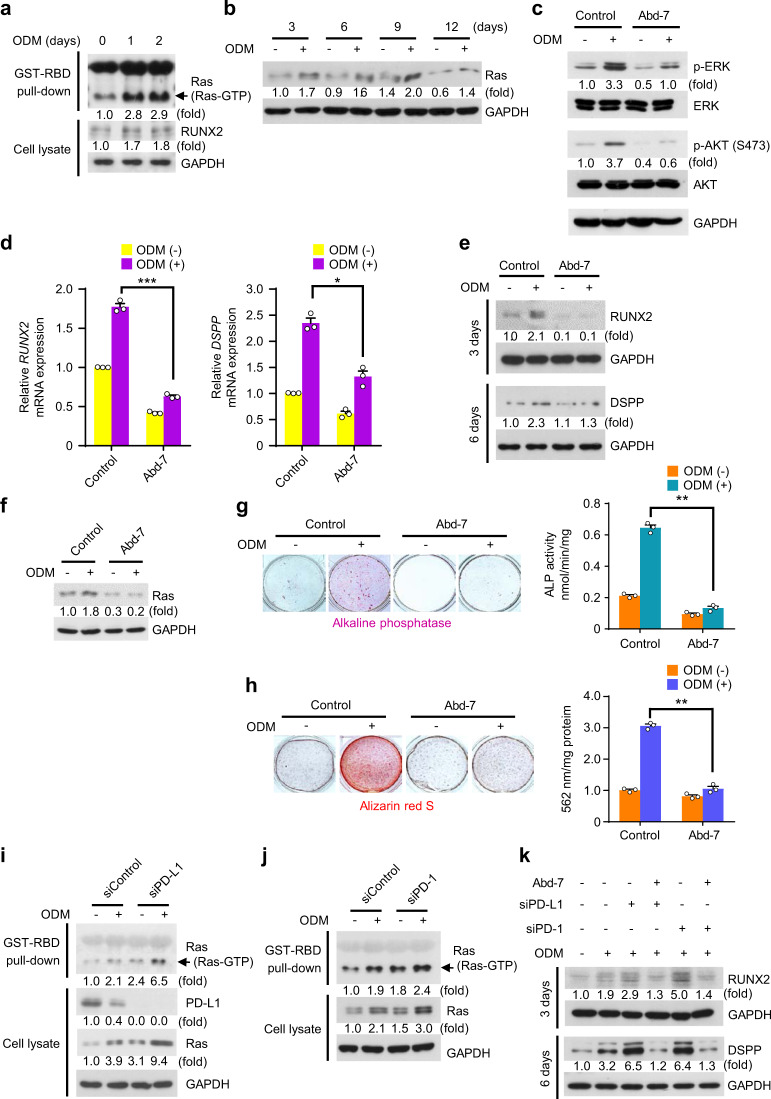
Silencing of PD-L1/PD-1 promotes hDPSCs differentiation into the osteo-/odontogenic lineage *via* Ras activation. **a** hDPSCs were cultured with or without ODM for the indicated days. RBD pull-down assay and immunoblotting analyses were carried out, and representative band intensity was quantified. **b** hDPSCs were cultured with or without ODM for the indicated days. Immunoblotting analyses were carried out and representative band intensity was quantified. **c** hDPSCs were cultured with or without ODM or Abd-7 (20 μmol·L^−1^) for one day. Immunoblotting analyses were carried out and representative band intensity was quantified. **d** and **e** hDPSCs were cultured with or without ODM or Abd-7 (20 μmol·L^−1^) for 3 days (for RUNX2) or 6 days (for DSPP). Real-time PCR data for *RUNX2* and *DSPP* (**d**). Immunoblotting analyses were carried out and representative band intensity was quantified (**e**). *P < 0.05; ***P < 0.001, two-way ANOVA test. **f** hDPSCs were cultured with or without ODM or Abd-7 (20 μmol·L^−1^) for 2 days. Immunoblotting analyses were carried out and representative band intensity was quantified. **g** and **h** hDPSCs were cultured with or without ODM or Abd-7 (20 μmol·L^−1^) for 6 days (for ALP staining and activity assay) or 21 days (for Alizarin red S staining and quantification). ALP staining and activity assay were performed (**g**). Alizarin red S staining was performed and quantified (**h**). **P < 0.01, two-way ANOVA test. **i** The control siRNA or PD-L1 siRNA-transfected hDPSCs were cultured with or without ODM for one day. RBD pull-down assay and immunoblotting analyses were carried out, and representative band intensity was quantified. **j** The control siRNA or PD-1 siRNA-transfected hDPSCs were cultured with or without ODM for one day. RBD pull-down assay and immunoblotting analyses were carried out, and representative band intensity was quantified. **k** The control siRNA, PD-L1 siRNA, or PD-1 siRNA-transfected hDPSCs were cultured with or without ODM or Abd-7 (20 μmol·L^−1^) for 3 days (for RUNX2) or 6 days (for DSPP). Immunoblotting analyses were carried out and representative band intensity was quantified.

As ODM-activated ERK and AKT signals were simultaneously induced by knockdown of either PD-L1 or PD-1, we were inspired to explore whether PD-L1/PD-1 signaling regulates the upstream effector of ERK and AKT. It has been reported that that GTP-bound Ras protein activates its prominent downstream signals-RAF/MEK/ERK or PI3K/AKT in response to numerous upstream stimuli.^15^ We first investigated Ras activity and its expression during osteo-/odontogenic differentiation of hDPSCs. Ras-binding domain (RBD) pull-down assay showed that ODM stimulation greatly induced Ras enzyme activity, which is a GTP-bound form, compared to their unstimulated control (Fig. 4a). In addition, total Ras protein expression levels were increased during osteo-/odontogenic differentiation of hDPSCs (Fig. 4b). Three Ras isoforms are expressed and upregulated by ODM stimulation in hDPSCs (Fig. S3). Next, we evaluated the role of enhanced Ras activity in the hDPSCs differentiation into the osteo-/odontogenic lineage. As expected, treatment with Ras inhibitor Abd-7^18,19^ greatly inhibited ODM-induced phosphorylation levels of ERK and AKT in hDPSCs (Fig. 4c). In addition, Abd-7 treatment resulted in decreased ODM-induced expression of RUNX2 and DSPP (Fig. 4d, e). Of note, the ODM-induced Ras expression was reduced by inhibition of Ras activation (Fig. 4f). Ultimately, Abd-7 treatment substantially reduced ODM-induced ALP activities (Fig. 4g) and ability to form mineralized nodules (Fig. 4h). These results demonstrate that Ras activity increases, which plays a vital role in hDPSCs differentiation into the osteo-/odontogenic lineage.

Earlier, we showed that knockdown either PD-L1 or PD-1 increased ODM-induced phosphorylation of ERK and AKT and those of -dependent osteo-/odontogenic differentiation. Therefore, we evaluated whether PD-L1/PD-1 functions through regulating Ras activity. RBD pull-down assay showed that the PD-L1 or PD-1 knockdown of hDPSCs significantly enhanced both basal and ODM-induced Ras enzyme activity compared to control hDPSCs (Fig. 4i, j). Moreover, the Ras activity-dependent Ras expression was increased by depletion of PD-L1 or PD-1 (Fig. 4i, j). To investigate whether PD-L1/PD-1 silencing-induced Ras activation is responsible for promoting the osteo-/odontogenic differentiation of hDPSCs, we treated the PD-L1 or PD-1 knockdown hDPSCs with Ras inhibitor Abd-7, and showed that PD-L1/PD-1 silencing-induced expression levels of differentiation markers were abolished by Ras inhibition (Fig. 4k). Overall, these data demonstrate that PD-L1 and PD-1 negatively regulate hDPSCs differentiation into the osteo-/odontogenic lineage *via* suppression of Ras activity.

### PD-L1/PD-1 signaling blockade nivolumab promotes hDPSCs differentiation into the osteo-/odontogenic lineage

**Fig. 5:**
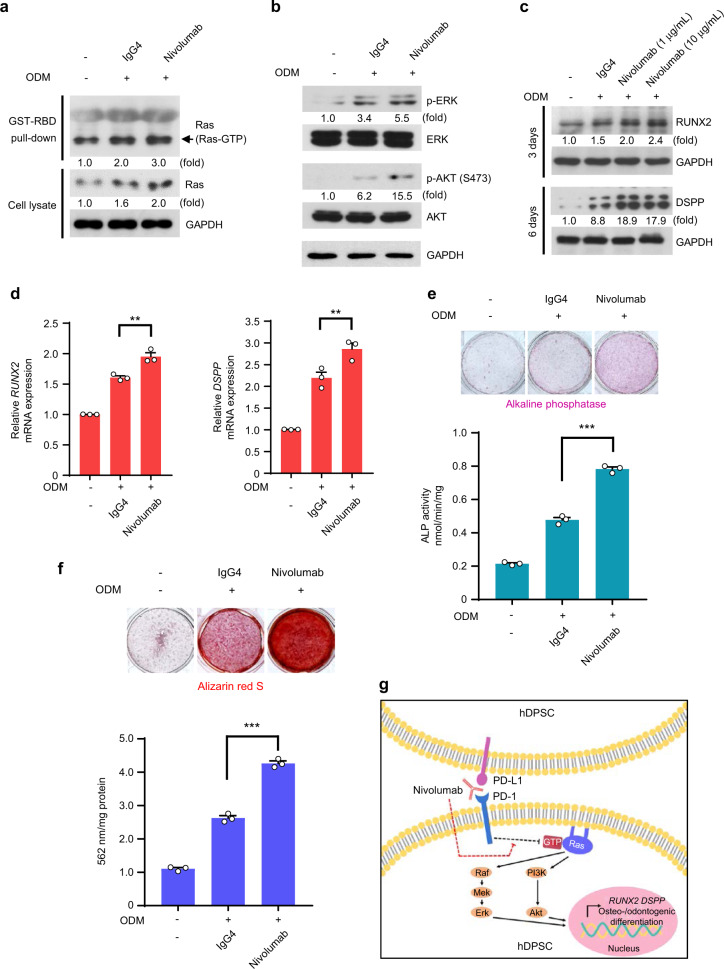
PD-L1/PD-1 signaling blockade nivolumab promotes hDPSCs differentiation into the osteo-/odontogenic lineage. **a** hDPSCs were cultured with or without ODM for one day in the presence of IgG4 or Nivolumab (10 μg/mL). RBD pull-down assay and immunoblotting analyses were carried out, and representative band intensity was quantified. **b** hDPSCs were cultured with or without ODM for one day in the presence of IgG4 or Nivolumab (10 μg·mL^−1^). Immunoblotting analyses were carried out and representative band intensity was quantified. **c** and **d** hDPSCs were cultured with or without ODM for 3 days (for RUNX2) or 6 days (for DSPP) in the presence of IgG4 or Nivolumab (1 or 10 μg·mL^−1^). Immunoblotting analyses were carried out and representative band intensity was quantified (**c**). Real-time PCR data for *RUNX2* and *DSPP* (**d**). **P < 0.01, Student’s *t* test. **e** and **f** hDPSCs were cultured with or without ODM for 6 days (for ALP staining and activity assay) or 21 days (for Alizarin red S staining and quantification) in the presence of IgG4 or Nivolumab (10 μg·mL^−1^). ALP staining and activity assay were performed (**e**). Alizarin red S staining was performed and quantified (**f**). ***P < 0.001, Student’s *t* test. **g** Schematic diagram of the proposed mechanism.

Based on our findings that PD-1 and PD-L1 had similar effects on Ras activity and its-dependent downstream signals and osteo-/odontogenic differentiation, we hypothesized that hDPSCs-intrinsic PD-1 signaling is engaged by PD-L1 to regulate osteo-/odontogenic differentiation and the corresponding signaling pathways. To confirm this, we used the FDA-approved nivolumab (Opdivo), to block PD-L1-mediated intrinsic PD-1 signaling.^13^ We treated hDPSCs with nivolumab or isotype control antibody in the presence of ODM, and revealed that nivolumab-treated hDPSCs had higher activity and expression of Ras (Fig. 5a), phosphorylation levels of ERK and AKT (Fig. 5b), and expression levels of differentiation markers (Fig. 5c, d) compared to the IgG4 control antibody-treated cells. As a result, the nivolumab-treated cells exhibited increased ALP activities (Fig. 5e) and ability to form mineralized nodules (Fig. 5f). These findings indicate that nivolumab-mediated hDPSCs-PD-1 blockade directly enhances osteo-/odontogenic differentiation, and further confirm that PD-L1-mediated hDPSCs-intrinsic PD-1 signaling negatively regulate osteo-/odontogenic differentiation through downregulation of Ras activation.

## Discussion

Many types of dental stem cells have been identified. These stem cells include the dental pulp stem cells (DPSCs),^1^ stem cells from exfoliated deciduous teeth (SHED),^20^ periodontal ligament stem cells (PDLSCs),^21^ gingiva-derived MSCs (GMSCs),^22^ apical papilla stem cells (APSCs),^23^ and stem cells from dental follicles (DFSCs).^24^ DPSCs are ectoderm-derived stem cells, which are originated from migrating neural crest cells.^25,26^ DPSCs share many biological characteristics, such as a fibroblast-like morphology, surface marker expression, differentiation, proliferation, and colony-forming behavior similar to those of MSCs, including bone marrow MSCs (BMMSCs) and adipose tissue-derived stem cells (ADSCs).^25,26^ However, their proliferation potential and differentiation potential varies; DPSCs have a higher proliferation rate and clonogenic potential than MSCs^1,27^ DPSCs exhibit stronger odontogenesis and neurogenesis capabilities, but relatively low potential to produce osteogeneic, adipogeneic, and chondrogeneic tissues than BMMSCs.^25,27^ In addition to their potential for proliferation and multilineage differentiation capacities, DPSCs have been shown to possess potent immunosuppressive activities^28,29^ that are found in BMMSCs.^29,30^

PD-1-mediated inhibitory signals play a critical role in immune tolerance and homeostasis. PD-1 signaling has been intensively studied with a focus on the PD-1-expressed immune cells, including activated T cells.^8^ Recently, functional roles of PD-1 signaling have been extended to non-immune cell types, such as tumor cells,^31–33^ retinal ganglion cells,^34^ and stem cells.^35^ In particular, Shi group demonstrated that SHED express PD-1, which regulates cell proliferation and differentiation.^35^ However, the potential functions and expressional patterns of PD-L1 and PD-1 during the differentiation of hDPSCs into the odontogenic lineage are unknown.

In this study, we found out that hDPSCs constitutively express both PD-L1 and PD-1 in the cytomembrane. However, only PD-L1 expression was lost upon initiation of osteo-/odontogenic differentiation, indicating that the PD-L1 expression may be unnecessary and could exert an inhibitory role in the hDPSCs differentiation into the osteo-/odontogenic lineage; when we depleted PD-L1 expression in hDPSCs, more hDPSCs differentiated into the osteo-/odontogenic lineage, demonstrating that PD-L1 expression negatively regulates the hDPSCs differentiation into the osteo-/odontogenic lineage. Although PD-1 expression was not altered during the hDPSCs differentiation into the osteo-/odontogenic lineage, PD-1 expression exhibited the same function as its ligand, PD-L1. Notably, nivolumab treatment, which prevents the binding of PD-L1 to PD-1,^13^ enhanced hDPSCs differentiation into osteo-/odontogenic lineage. These data suggest that the hDPSCs-intrinsic function of the PD-L1/PD-1 axis plays an inhibitory role in the osteo-/odontogenic differentiation.

Most defined PD-1 signaling has been demonstrated by studies on acutely activated T cells. In T-cell receptor (TCR)-stimulated T cells, ligands-engaged PD-1 becomes phosphorylated at two tyrosine residues in its cytoplasmic domain, leading to binding of protein tyrosine phosphatases (PTPs), such as SHP2.^7^ These PTPs directly dephosphorylate proximal TCR signaling molecules and subsequently antagonize downstream signaling of the TCR, such as PI3K/AKT and ERK.^9^ Notably, the inhibitory roles of PD-1 in the AKT and ERK signals were also observed in some specific tumor cells.^33^ Consistently, our study revealed that PD-1 signaling suppresses the pro-osteo-/odontogenic differentiating pathways, AKT and ERK, in hDPSCs. Interestingly, we found that Ras enzyme activity is induced; this is required for its own expression, the activation of both ERK and AKT pathways, and hDPSCs differentiation into osteo-/odontogenic lineage. To the best of our knowledge, this is the first study to show the role of Ras protein in hDPSCs differentiation into the odontogenic lineage. Nivolumab-mediated hDPSCs-PD-1 blockade and hDPSCs-specific PD-L1 or PD-1 knockdown studies showed that hDPSCs-intrinsic PD-1 signaling negatively regulates Ras enzyme activity and its dependent Ras expression. On the other hand, hDPSCs-intrinsic PD-1 silencing-induced osteo-/odontogenic differentiation was reversed by pharmacologic inhibition of Ras, suggesting that PD-1 signaling inhibits osteo-/odontogenic differentiation through deactivation of Ras enzyme activity. The divergent effects of PD-1 signaling on Ras activity have been reported in different cell types. For instance, PD-1 inhibits TCR-mediated activation of Ras through an unknown mechanism in T cells,^36^ in contrast; PD-1 signaling enhances Ras activation through phosphatase activity of SHP2 in tumor cells.^37,38^ In addition to SHP2 phosphatase activity, it functions as an adapter that binds to receptor tyrosine kinases (RTKs) and recruits the GRB/SOS complex to the plasma membrane, enhancing GTPase-activating protein SOS-mediated Ras activation.^39^ SHP2 is also expressed by hDPSCs.^35^ However, there is a need for further investigations on whether SHP2 is directly or indirectly involved in the Ras activation for hDPSCs differentiation into osteo-/odontogenic lineage. In addition, further studies are needed for clear elucidation of the precise underlying mechanism of PD-1-mediated Ras inactivation in hDPSCs.

hDPSCs is from human adult permanent dental pulp tissue^1^ while SHED are isolated from exfoliated deciduous teeth.^20^ These cells have abilities in multilineage differentiation, self-renewal, and immunomodulatory functions.^40^ Hence, both hDPSCs and SHED have potential advantages in regenerative treatments and immunotherapy. Compared with hDPSCs, SHED show a higher differentiation potential, proliferation, and ability to form mineralized nodules in vivo.^20,40–42^ Liu et al. showed that PD-1 negatively regulates the SHED differentiation into osteo-/odontogenic lineage through suppressing β-catenin signal pathway,^35^ which is a distinct mechanism from our findings, although the role of PD-1 in the differentiation is the same in hDPSCs and SHED.

We showed different expressional patterns of PD-L1 and PD-1 during the hDPSCs differentiation into osteo-/odontogenic lineage. In our differentiation system using general differentiation medium, only PD-L1 expression was downregulated during osteo-/odontogenic differentiation. It was revealed that dexamethasone inhibits *CD274* transcriptional expression, but has no effect on *PDCD1*, in hDPSCs. Consistent with our results, dexamethasone has been shown to mediate transcriptional suppression of *CD274* depending on the GR/STAT3 complex.^43^ These results suggest that the regulatory molecular mechanisms in the PD-L1/PD-1 expression seem to be different at the transcriptional levels.

Nivolumab is the most representative PD-1 inhibitor for the clinical treatment of advanced tumors.^10–13^ Besides cancer immunotherapy, as the first study demonstrating the effect of nivolumab in promoting hDPSCs differentiation into the osteo-/odontogenic lineage, the present study findings have strong clinical positive implications in the field of dental pulp therapy and provides a foundation for future clinical trials in dental pulp immunotherapy; our in vitro findings provide a basis for further research on the nivolumab effects with preclinical animal models.

In conclusion, we showed that inhibition of hDPSCs-intrinsic PD-L1/PD-1 signaling promotes osteo-/odontogenic differentiation *via* Ras activation (Fig. 5g). Moreover, for the first time, we showed that FDA-approved PD-1 blockade nivolumab exerted a pro-osteo-/odontogenic differentiating effect on hDPSCs. Therefore, a combination of hDPSCs transplantation and PD-1 blockade or Ras activation could be a potential new therapeutic method for the regeneration or repairment of the dentin-pulp tissue.

## Materials and methods

### Chemicals and reagents

Abd-7 (Cat. # HY-122862) was purchased from MedChem Express (Princeton, NJ). l-ascorbic acid (Cat. # A4544), β-glycerophosphate (Cat. # 50020), dexamethasone (Cat. # D1756), ALP buffer (Cat. # A9226), p-Nitrophenyl phospahte tablets (Cat. # P5744), Alizarin Red (Cat. # TMS-008), hexadecylpyridinium chloride monohydrate (Cat. # C9002), and U0126 (Cat. # 662005) were acquired from Sigma-Aldrich (St. Louis, MO). MK-2206 (Cat. # S1078) was procured from Selleck Chemicals (Houston, TX). SB203580 (Cat. # S-3400) and PD98059 (Cat. # P-4313) were purchased from LC Laboratories (Woburn, MA). Nivolumab (Cat. # SY946414944) was acquired from SY innovation (Pyeongtaek, Korea). Ultra-LEAF™ purified human IgG4 isotype control antibody (Cat. # 403701) was acquired from BioLegend (San Diego, CA). Various antibodies were procured: PD-L1 (Cat. # 13684 S), PD-1(Cat. # 86163S), ERK (pT202/pY204, Cat. # 9101), ERK (Cat. # 9102), AKT (pS473, Cat. # 4060), and AKT (Cat. # 9272) were from Cell Signaling Technology (Danvers, MA); tubulin (Cat. # T6074) was from Sigma-Aldrich (St. Louis, MO); and E-cadherin (Cat. # sc-8426), DSPP (Cat. # sc-73632), RUNX2 (Cat. # sc-101145), and GAPDH (Cat. # sc-47724) were from Santa Cruz Biotechnology (Santa Cruz, CA). The StemAb Alkaline Phosphatase Staining Kit II (Cat. # 00-0055) was purchased from ReproCell (Beltsville, MD).

### Culture of hDPSCs

hDPSCs (Cat. # PT-5025; Switzerland) were maintained in α-MEM (Welgene, South Korea), supplemented with 10% FBS (MERCK; Kenilworth, NJ). The passage numbers of the cells used in this study were limited to 2–3. To induce the hDPSCs differentiation into the osteo-/odontogenic lineage, the cells were cultured with osteo-/odontogenic differentiation medium (ODM) containing l-ascorbic acid (50 µg·mL^−1^), β-glycerophosphate (10 mmol·L^−1^), and dexamethasone (10 µmol·L^−1^).

### Real-time PCR analysis

Real-time PCR was performed as described our previous study.^17^ The sequences of primer pairs were: *RUNX2* 5′-CCACTGAACCAAAAAGAAATCCC-3′ and 5′- GAAAACAACACATAGCCAAACGC-3′; *DSPP* 5′-ATATTGAGGGCTGGAATGGGGA-3′ and 5′-TTTGTGGCTCCAGCATTGTCA-3′; *KRAS* 5′-GAGGCCTGCTGAAAATGACTG-3′ and 5′-ATTACTACTTGCTTCCTGTAGG-3′; *NRAS* 5′-GAGTTACGGGATTCCATTCATTGAAAC-3′ and 5′-TGGCGTATTTCTCTTACCAGTGTGTAAAA-3′; *HRAS* 5′-TACGGCATCCCCTACATCGAGAC-3′ and 5′-CACCAACGTGTAGAAGGCATCCTC-3′; *PDCD1* 5′-GACAGCGGCACCTACCTCTGTG-3′ and 5′-GACCCAGACTAGCAGCACCAGG-3′; *CD274* 5′-GTGGCATCCAAGATACAAACTCAA-3′ and 5′-TCCTTCCTCTTGTCACGCTCA-3′; and *GAPDH* 5′-GCATCTTCTTTTGCGTCG-3′ and 5′-TGTAAACCATGTAGTTGAGGT-3′.

### Immunoblot analysis

Immunoblot analysis was performed in accordance to our previous study.^44^ The used antibodies were: rabbit anti-PD-L1 (1:1000), rabbit anti-PD-1(1:1000), rabbit anti-ERK (pT202/pY204, 1:1000), rabbit anti-ERK (1:1000), rabbit anti-AKT (pS473, 1:1000), rabbit anti-AKT (1:1000), mouse anti-E-cadherin (1:500), mouse anti-DSPP (1:500), mouse anti-RUNX2 (1:500), mouse anti-GAPDH (1:500), and mouse anti-tubulin (1:3000). The ImageJ 1.53e software (National Institutes of Health) was used for quantification of band intensity. All immunoblotting experiments were repeated three times. The original uncut blots are displayed in Fig. S4.

### Cell fractionation

hDPSCs cultured in 10 cm plate were scraped and then spun down *via* centrifugation (700 g per 5 min). Cells were homogenized using TNE buffer (50 mmol·L^−1^ Tris–HCl, 150 mmol·L^−1^ NaCl, 1 mmol·L^−1^ EDTA) supplemented with 0.5% Brij 58. The supernatant was collected *via* centrifugation (700 g per 10 min) and then transferred to new centrifuge tubes and centrifuged (10 000 g per 30 min). The cytoplasm was collected from the supernatant. The pellet was resuspended in TNE buffer supplemented with 0.5% Brij 58, 60 mmol·L^−1^ n-octyl-β-d-glucopyranoside, and 0.2% deoxycholic acid for 1 h on ice and centrifuged (12 000 g per 20 min). The membrane was collected from the supernatant.

### siRNA transfection

PD-L1 siRNA (Cat. # 4392420) and PD-1 siRNA (Cat. # AM16708) were purchased from Thermo Fisher (Pittsburgh, PA). Transfection of the siRNA in hDPSCs was carried out using Lipofectamine^TM^ RNAiMAX (Cat. # 13778, Thermo Fisher; Pittsburgh, PA) based on the manufacturer’s protocols.

### RAF-Ras binding domain (RBD) pull-down assay

The Ras Pull-Down Activation Assay Biochem Kit (Cat. # BK008) was purchased from Cytoskeleton (S Acoma St Denver, CO), and Ras activity was assessed according to the manufacturer’s protocols. Briefly, cultured hDPSCs were lysed and the collected lysate was incubated with RAF-Ras binding domain (RBD) beads. Ras was detected by immunoblot analysis with an anti-Ras antibody. The detected Ras is regarded as the GTP-bound active form.

### ALP and alizarin red S staining

To induce osteo-/odontogenic differentiation, hDPSCs (1 × 10^5^ cells per well) were plated into 12-well plates coated with 10% gelatin, cultured until 100% confluent, then ODM was added for the indicated days. The medium was changed every third day. An ALP staining was performed as described our previous study.^17^ For an ALP activity assay, hDPSCs were dissolved with ALP buffer, and then an ALP activity was determined in cell lysate using *p*-Nitrophenyl phosphate tablets. Absorbance of supernatant was recorded at 405 nm. An Alizarin red S staining and quantification of calcium deposition were performed as described our previous study.^17^ The data for ALP activity and quantification of calcium deposition was normalized by the total protein concentration.

### Data analysis

All the quantitative results are presented as the means ± standard deviation (SD) of at least three independent experiments with duplicates or triplicates. A two-group comparison or a simultaneous comparison of more than two groups was conducted using a two-sided, two-sample Student’s *t*-test or two-way ANOVA with Sidak’s multiple comparisons test, respectively. *P* < 0.05 was considered statistically significant.

## Supplementary information

Supplemental information

## Data Availability

All data are included in the manuscript.
