# Supplementary material for: Blockade of PD-L1/PD-1 signaling promotes osteo-/odontogenic differentiation through Ras activation
Source: Int J Oral Sci. 2022 Apr 1;14:18. doi: 10.1038/s41368-022-00168-2 (PMC8976080; doi:10.1038/s41368-022-00168-2)

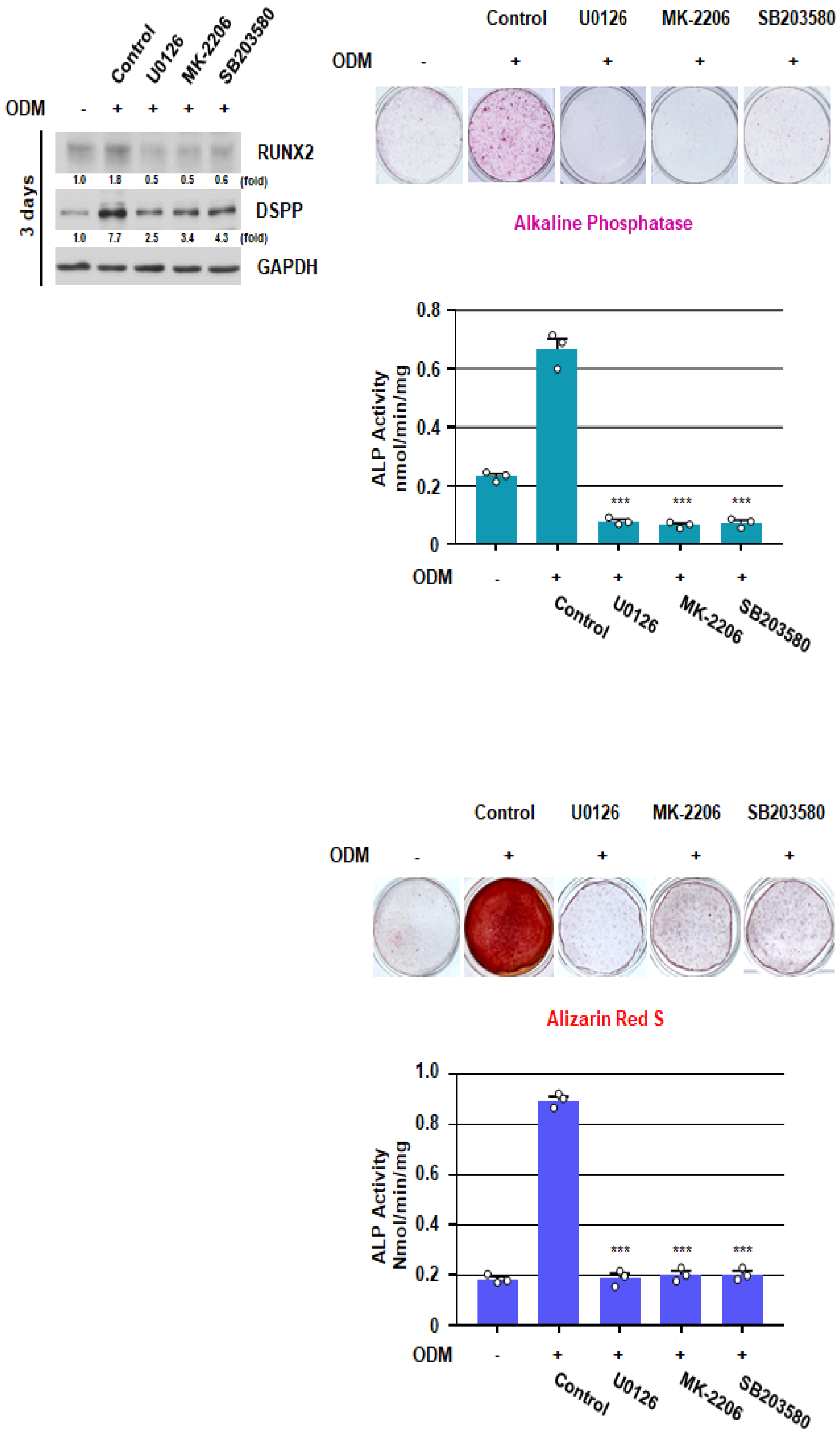

Fig. S1. Effects of ERK, AKT, and p38 pathways on osteo-/odontogenic differentiation of hDPSCs

hDPSCs were cultured with or without ODM in the presence or absence of U0126 (10 μM),

MK-2206 (5 μM), or SB203580 (10 μM). Immunoblotting analyses were performed with the indicated antibodies. ALP staining and ALP activity assay were performed. Alizarin red S staining was performed and quantified. Data represent the means ± s.d. of three independent experiments. ***$P < 0.001$, based on the Student's t test.

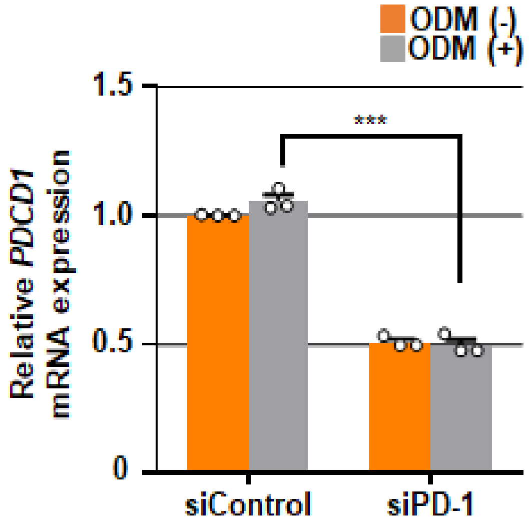

Fig. S2. Depletion of PD-1 by PD-1 siRNA in hDPSCs

hDPSCs were transfected with control siRNA or PD-1 siRNA, and then cultured with or without ODM for 1 day. Relative mRNA expression levels of *PDCD1* were determined by real-time PCR. Data represent the means ± s.d. of three independent experiments. ***$P< 0.001$, based on the Student's t test.

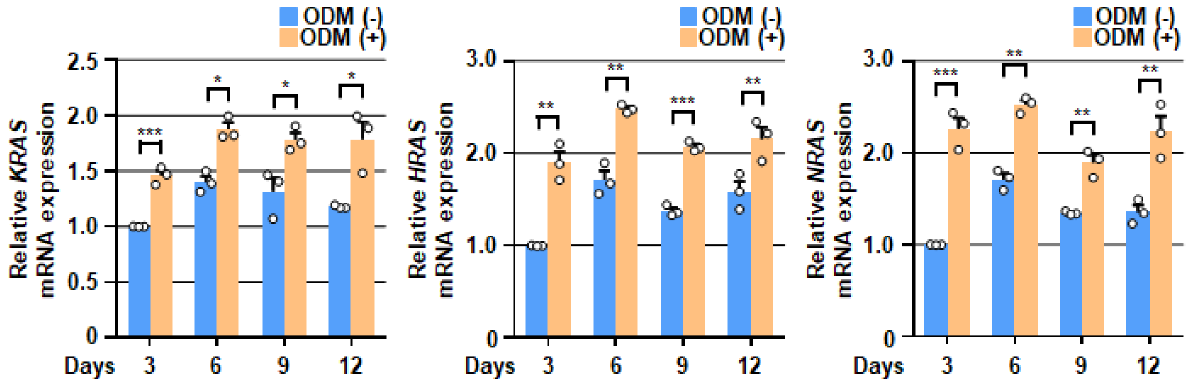

Fig. S3. Three Ras isoforms levels were upregulated during osteo-/odontogenic differentiation of hDPSCs

hDPSCs were cultured with or without ODM for the indicated days. Relative mRNA expression levels of *KRAS*, *HRAS*, and *NRAS* were determined by real-time PCR. Data represent the means ± s.d. of three independent experiments. **P*< 0.05; ***P*< 0.01; ****P*< 0.001, based on the Student's t test

Fig. S4

Fig. 1a

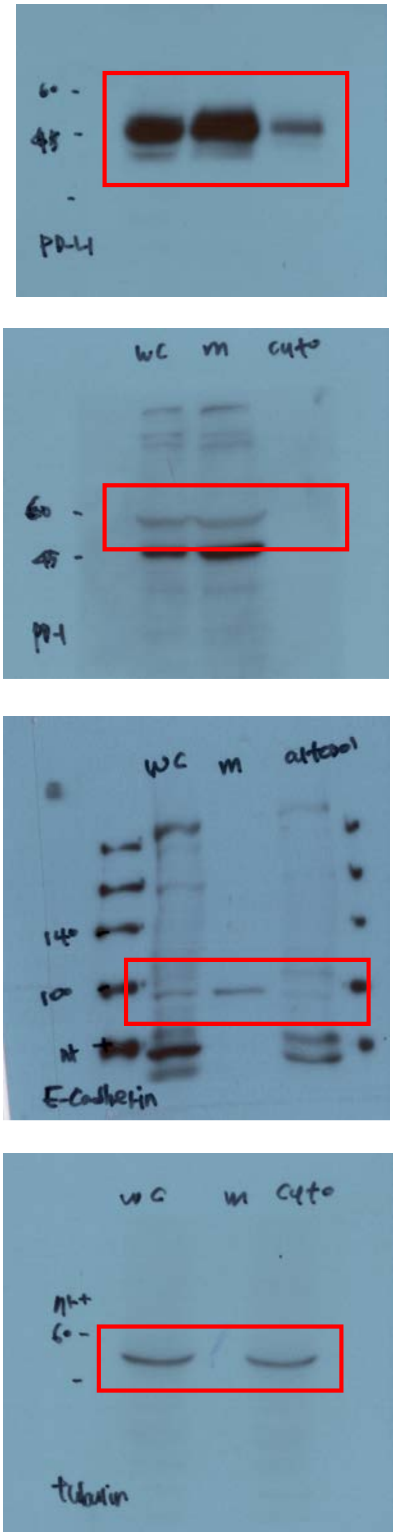

Fig. 1b

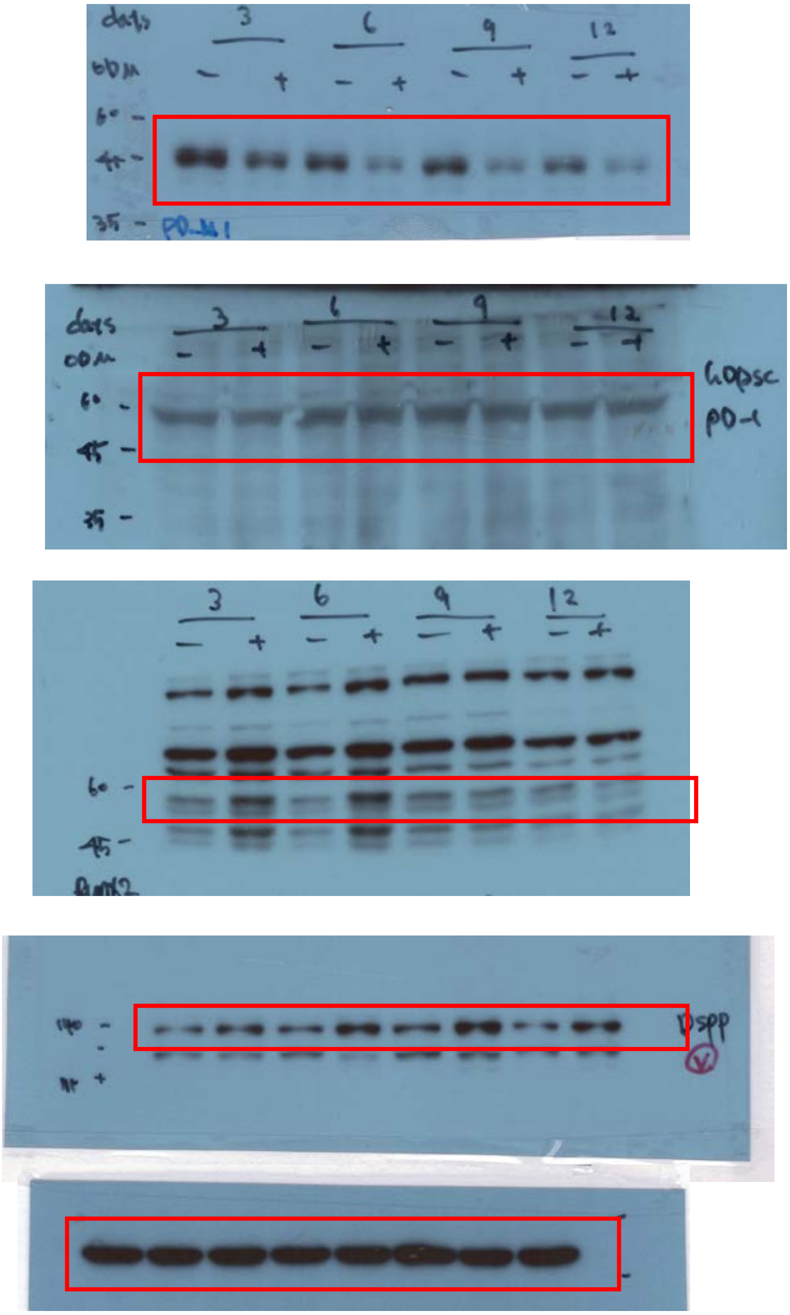

Fig. 1d

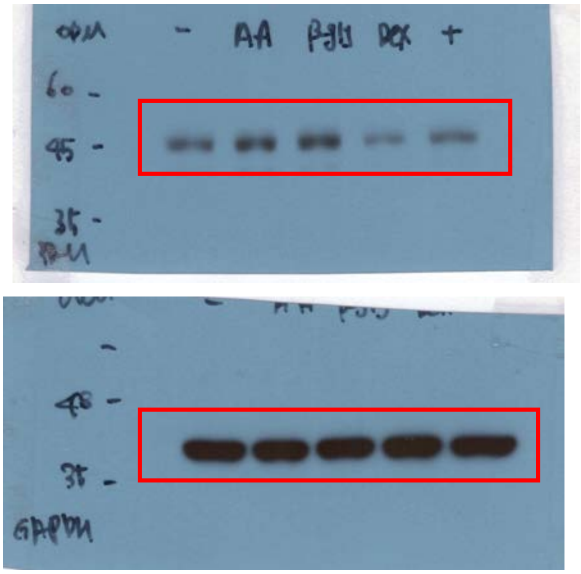

Fig. 2a

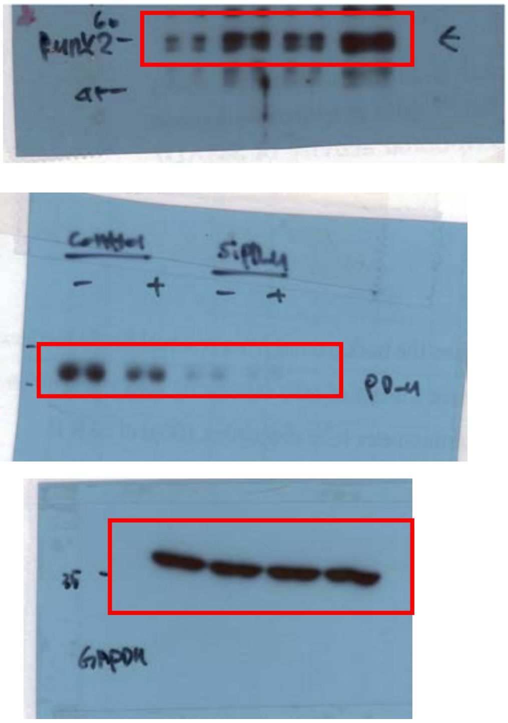

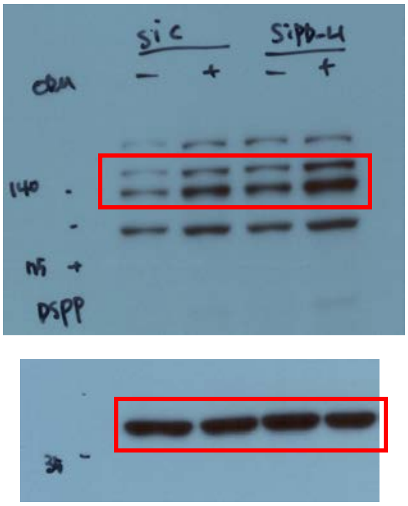

Fig. 2e

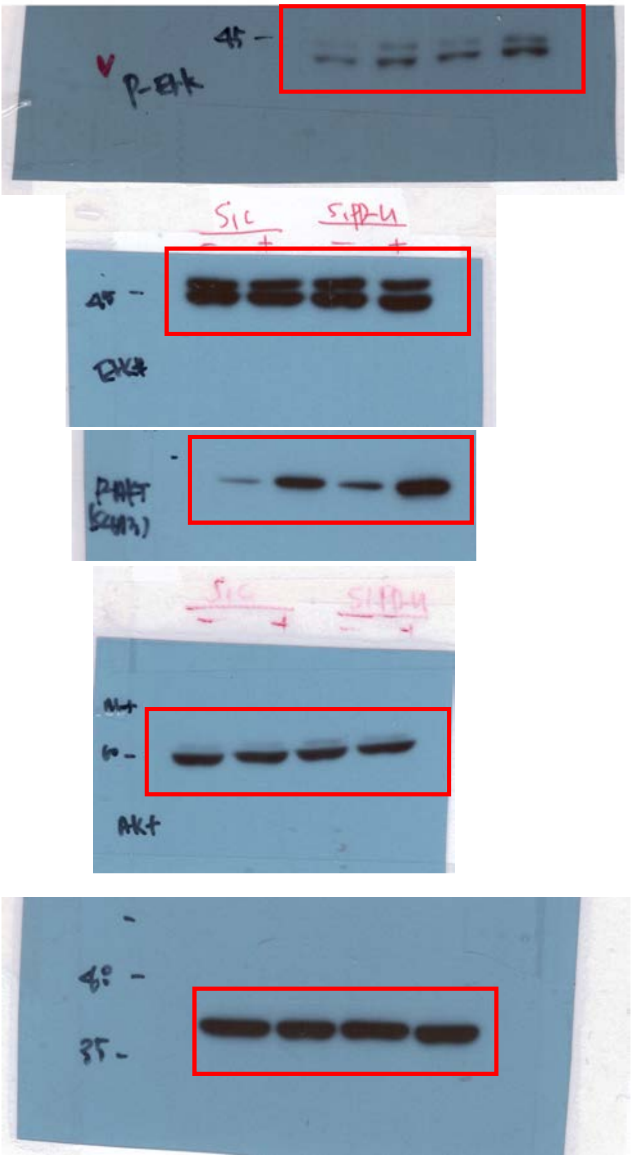

Fig. 2f

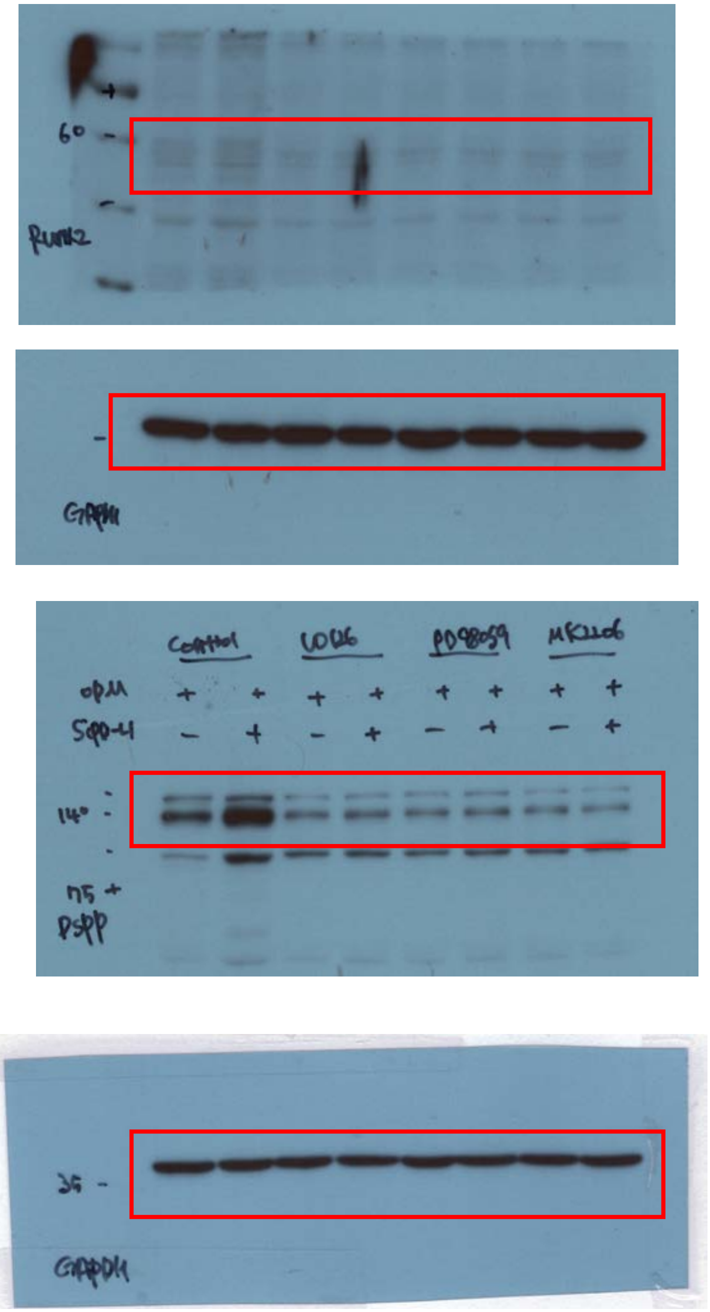

Fig. 3a

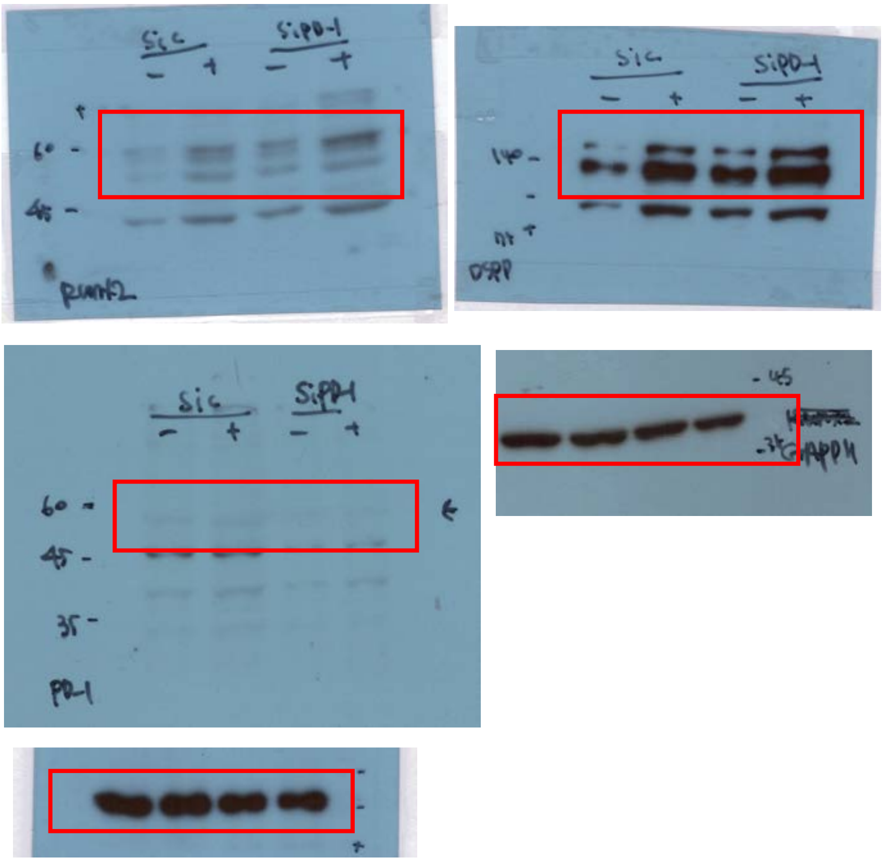

Fig. 3e

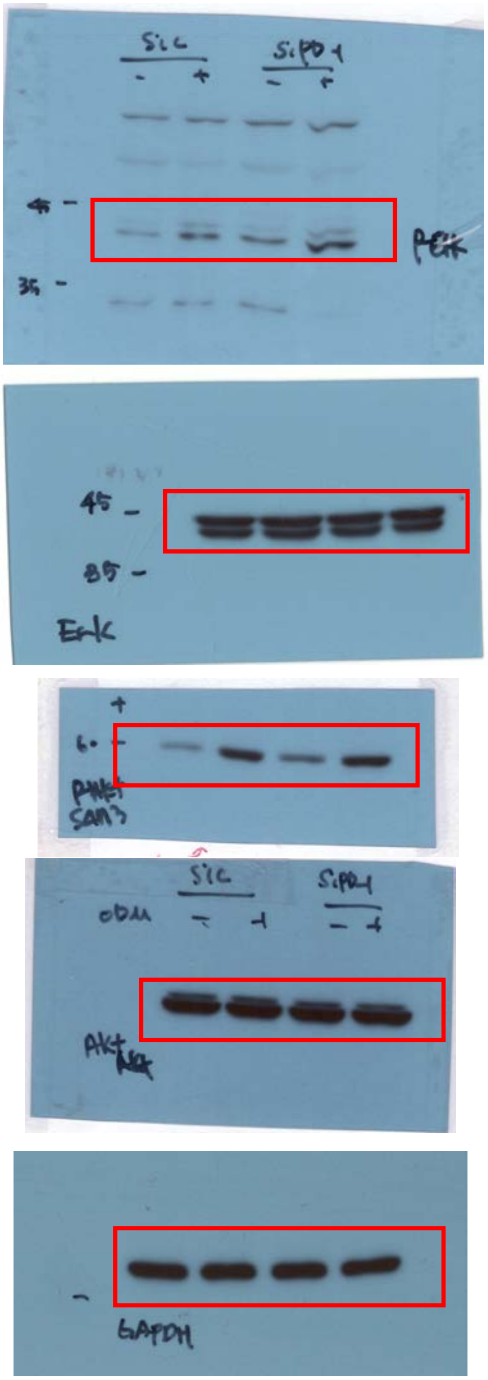

Fig. 3f

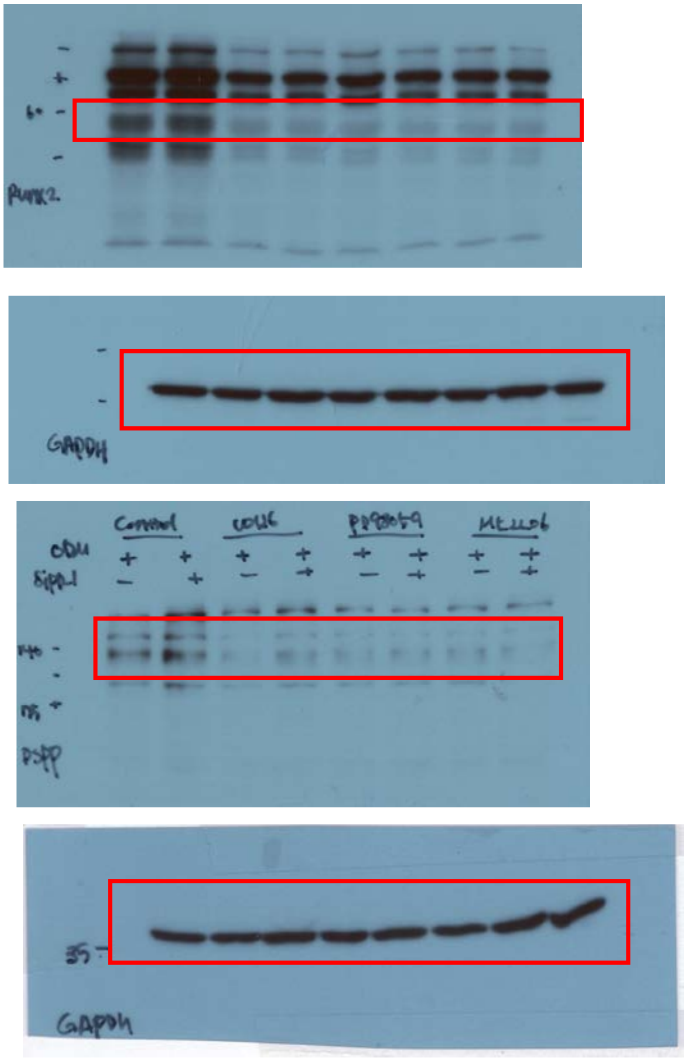

Fig. 4a

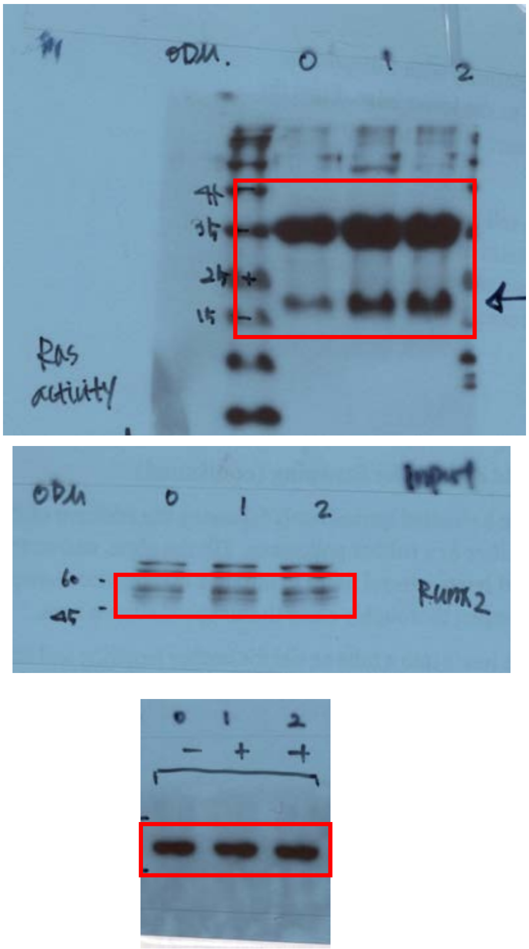

Fig. 4b

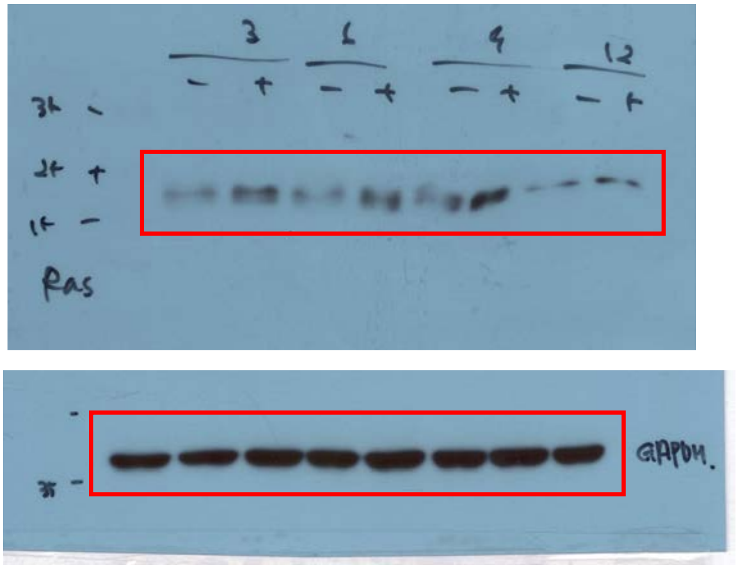

Fig. 4c

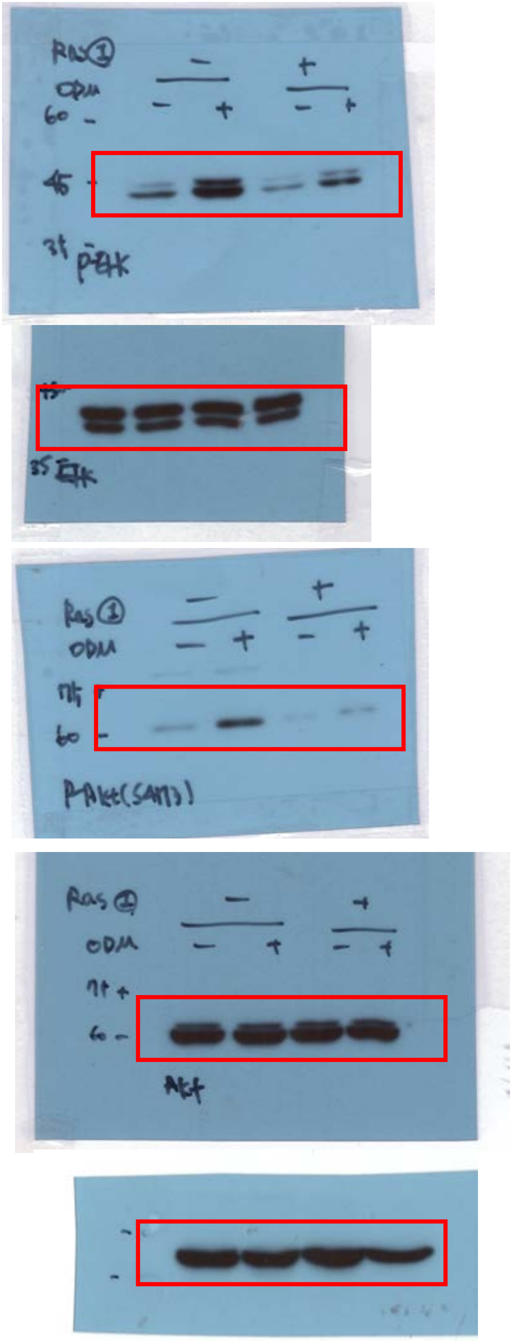

Fig. 4e

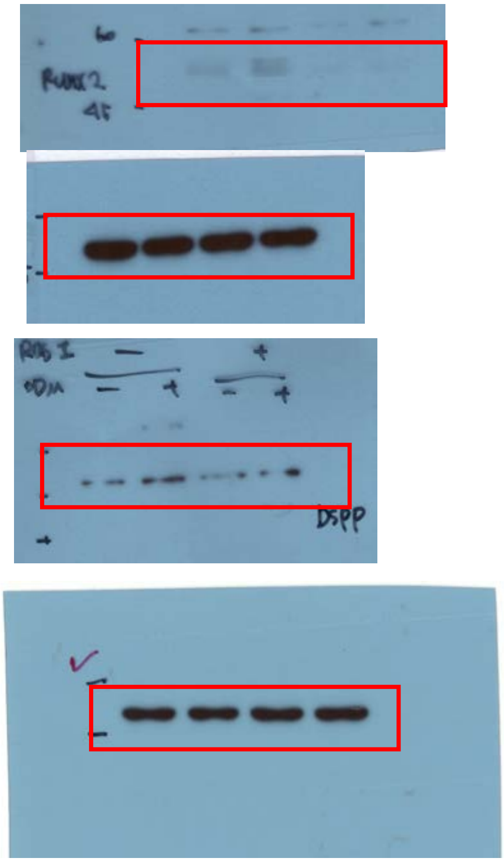

Fig. 4f

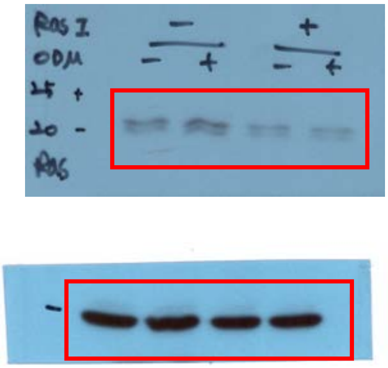

Fig. 4i

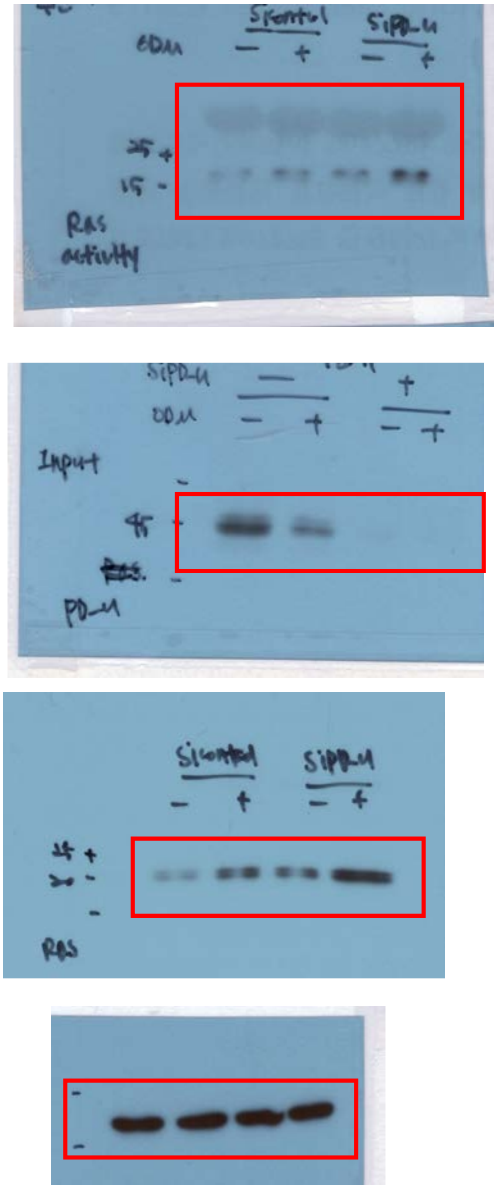

Fig. 4j

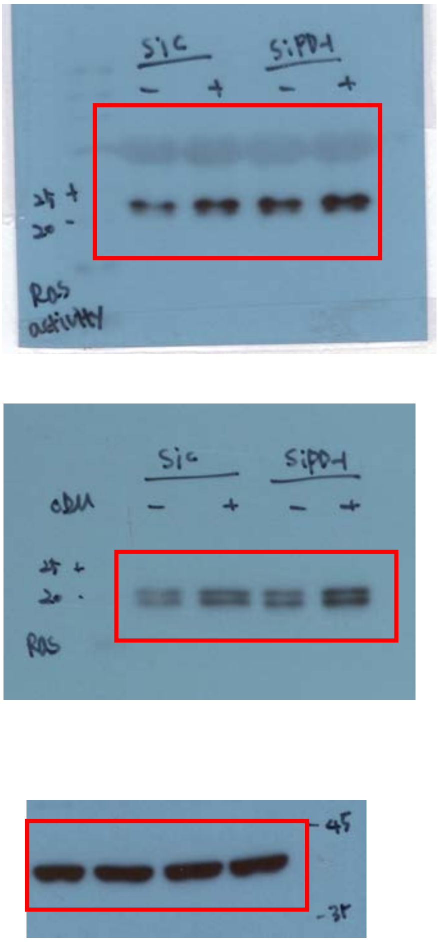

Fig. 4k

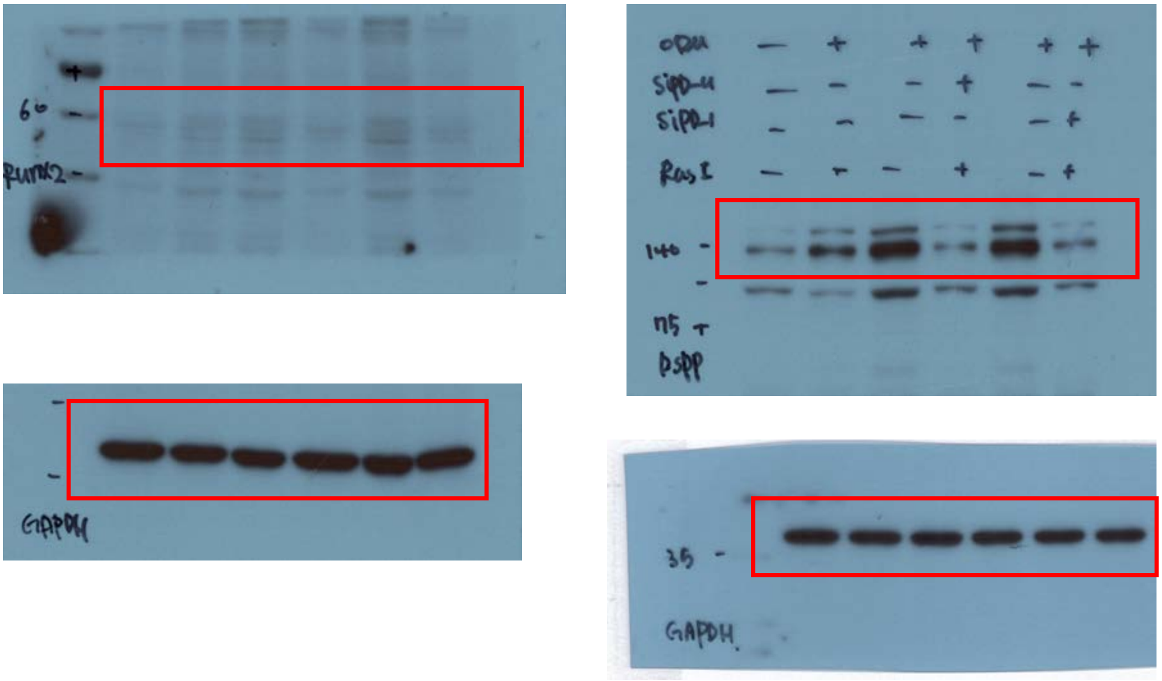

Fig. 5a

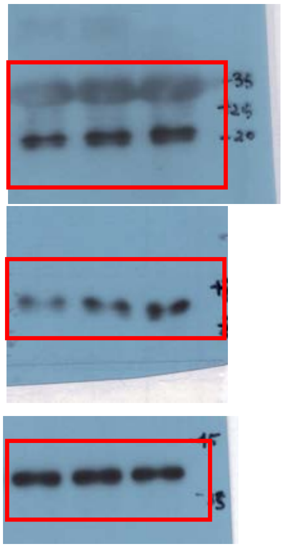

Fig. 5b

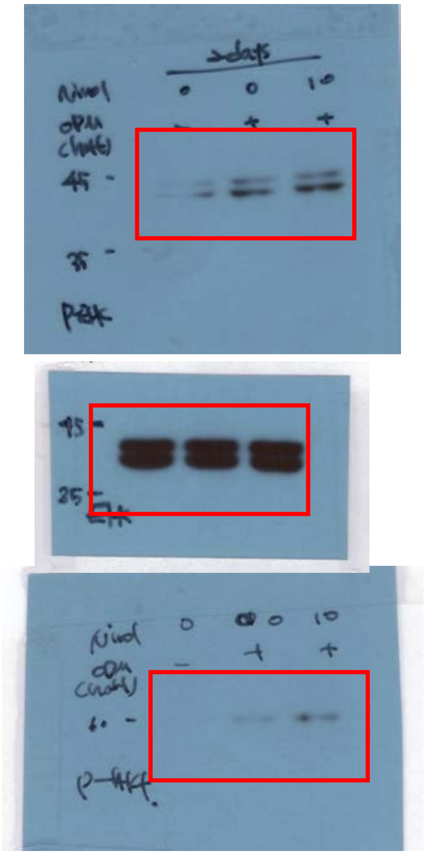

Figure 5C

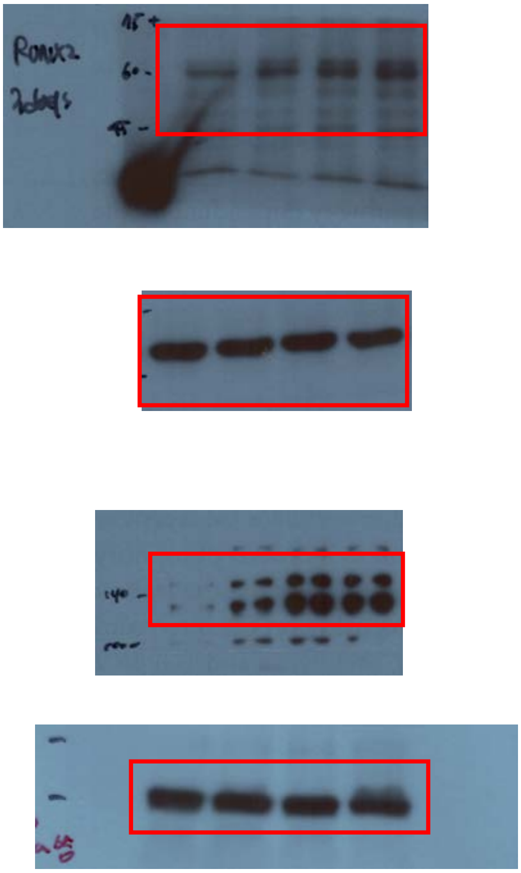

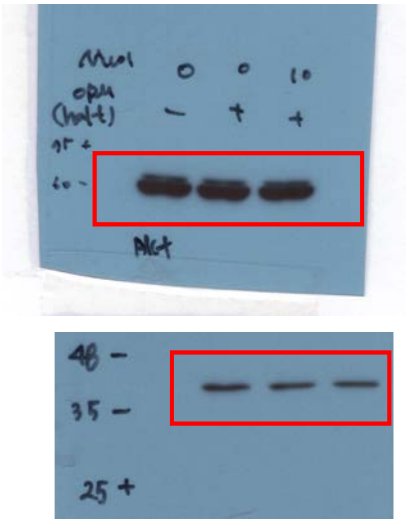

Fig. S1

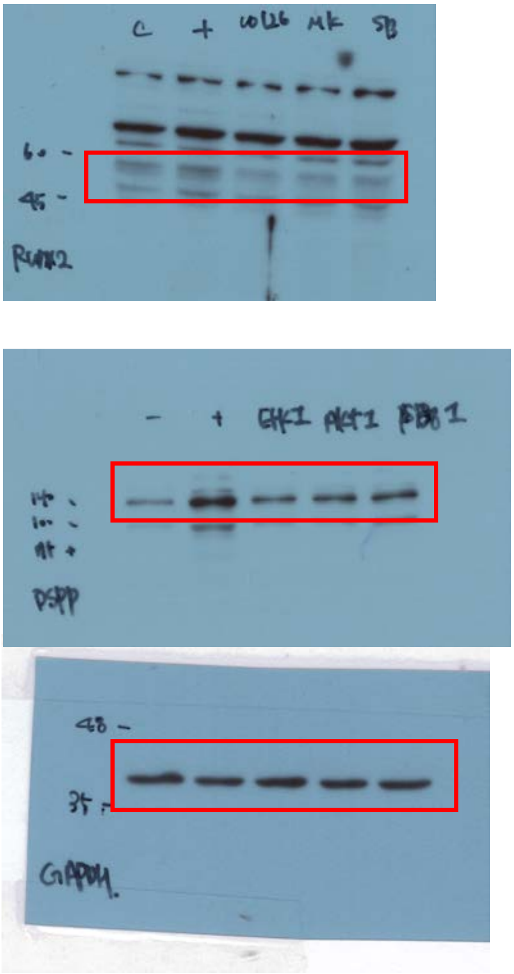

Supplement: Supplementary file 1 — Supplemental information [file 41368_2022_168_MOESM1_ESM.pdf]
